# Statistical Modeling of Humoral Immune Response Dynamics to mRNA COVID-19 Vaccines in Nursing Home Residents and Healthcare Workers from Southern Italy

**DOI:** 10.3390/v18010109

**Published:** 2026-01-14

**Authors:** Filippo Domma, Luca Soraci, Ersilia Paparazzo, Ilaria Amerise, Mirella Aurora Aceto, Teresa Serra Cassano, Dina Bellizzi, Salvatore Claudio Cosimo, Francesco Morelli, Andrea Corsonello, Giuseppe Passarino, Alberto Montesanto

**Affiliations:** 1 Department of Economics, Statistics and Finance “Giovanni Anania”, University of Calabria, 87036 Rende, Italy; filippo.domma@unical.it (F.D.); ilaria.amerise@unical.it (I.A.); 2 Unit of Geriatric Medicine, Italian National Research Center on Aging (INRCA IRCCS), 87100 Cosenza, Italy; l.soraci@inrca.it (L.S.); ersilia.paparazzo@unical.it (E.P.); a.corsonello@inrca.it (A.C.); 3 Department of Biology, Ecology and Earth Sciences, University of Calabria, 87036 Rende, Italy; mirellaaurora.aceto@unical.it (M.A.A.); teresa.serracassano@unifi.it (T.S.C.); dina.bellizzi@unical.it (D.B.); giuseppe.passarino@unical.it (G.P.); 4 Department of Statistic, Computer Science and Application, DiSIA, University of Florence, Viale Morgagni, 59, 50134 Florence, Italy; 5 SADEL S.p.A., 88836 Cotronei, Italy; salvatorecosimo@email.it (S.C.C.); morelli-f@libero.it (F.M.); 6 Department of Pharmacy, Health and Nutritional Sciences, University of Calabria, 87100 Cosenza, Italy

**Keywords:** nursing home residents, vaccination strategies, immunization, antibodies, COVID-19

## Abstract

Vaccination has been a cornerstone of the public health response to the COVID-19 pandemic, particularly in protecting older and frail populations. A detailed characterization of antibody titer dynamics and their determinants represents a crucial step toward optimizing vaccination strategies. However, antibody titers are bounded within assay-specific limited intervals and often display skewness and intra-subject correlation, which limit the suitability of conventional modeling approaches. We analyzed longitudinal antibody titer data from 608 residents and staff members of five nursing homes in Calabria (southern Italy) using beta-generalized linear mixed models (β-GLMMs). This framework enabled simultaneous modeling of the mean humoral response (μ), precision parameter (ϕ), and probability of achieving the maximum immune response (α), thereby providing a comprehensive assessment of factors influencing immune dynamics. Two distinct patterns of antibody titer evolution were identified. Among nursing home residents, stroke was associated with higher antibody concentrations, whereas atrial fibrillation, lower body mass index, non-Alzheimer’s dementia, and chronic obstructive pulmonary disease were linked to reduced responses. The β-GLMM approach allowed for a more accurate identification of demographic and clinical determinants compared with traditional methods. These findings underscore the utility of β-GLMMs for analyzing bounded longitudinal immunological data and highlight key factors shaping vaccine-induced immunity. Such insights may lead to more tailored immunization strategies in vulnerable older populations.

## 1. Introduction

The coronavirus disease 2019 (COVID-19) pandemic emerged at the end of 2019 and rapidly spread worldwide, initially affecting China and subsequently other countries, including South Korea and Italy, which experienced some of the earliest and most severe outbreaks outside Asia. By January 2020, China had reported more than 80,000 confirmed cases and over 3000 deaths, with early estimates of the basic reproduction number (R_0_) ranging between 2.2 and 3.6, indicating a high transmission potential [1]. Shortly thereafter, South Korea experienced one of the earliest large outbreaks outside China, exceeding 10,000 confirmed cases by March 2020, largely driven by superspreading events but with comparatively lower case fatality rates due to extensive testing and contact tracing strategies [2]. In early 2020, Italy became the epicenter of the outbreak in Europe, experiencing a rapid surge in cases, hospitalizations, and mortality that placed unprecedented pressure on the healthcare system. By March 2020, Italy had reported more than 100,000 confirmed cases and over 10,000 deaths, representing one of the highest mortality rates worldwide at that time [3].

The COVID-19 pandemic has posed an unprecedented global health challenge, leading to widespread morbidity, mortality, and socio-economic disruption [4,5,6]. Vaccination has emerged as the most effective public health intervention to control the spread of SARS-CoV-2, reduce severe disease, and prevent healthcare system overload, particularly in older and frail people [7].

In response to the global health emergency, unprecedented efforts were undertaken to develop effective vaccines against SARS-CoV-2. Vaccine development progressed at exceptional speed, particularly in the United States and China, leading to the rapid authorization of multiple vaccine platforms. Among these, the first widely deployed vaccine was the mRNA-based BNT162b2 (Pfizer–BioNTech), developed in the United States, which demonstrated high efficacy in preventing symptomatic infection [8,9]. Other vaccine platforms, including inactivated and live-attenuated or viral-vector-based vaccines, were also developed and implemented worldwide [10,11,12,13,14]. These vaccination strategies played a pivotal role in reducing severe disease, hospitalizations, and mortality, especially among older and clinically vulnerable populations. Extensive clinical and real-world evidence has demonstrated that timely and widespread vaccination not only decreased infection rates but also mitigated the impact of emerging variants [15,16,17]. In this context, mathematical modeling provided a robust framework to quantify and compare vaccination strategies, optimize resource allocation, and explore “what-if” scenarios, making it an indispensable component of the public health response to COVID-19 [15,16,17,18].

In numerous biomedical contexts, practitioners with limited data modeling expertise often remain unaware of the potential provided by appropriate statistical models for elucidating complex biological phenomena; consequently, they often employ methods, and in some cases models, that are unsuitable for interpreting the systems under investigation. Beta-generalized linear mixed models (β-GLMMs) constitute a class of statistical frameworks with considerable potential for applications in biomedical research. Β-GLMMs began to attract attention in biomedical research due to their ability to accommodate continuous outcomes constrained within the (0, 1) interval while accounting for both intra-subject correlation and outcome skewness. For instance, within this framework, Hunger et al. demonstrated that a β-GLMM provided superior likelihood-based fit and respected the boundedness of health-related quality-of-life scores collected longitudinally in older adults and stroke patients compared with traditional linear mixed models [19]. More recently, Di Brisco and Migliorati proposed an augmented β mixture mixed-effects model to handle constrained biomarkers and clinical scores with excess tail observations in long-term Parkinson’s disease cohorts, illustrating both improved robustness to outliers and enhanced interpretability of random effects [20].

Despite their undoubted advantages, the uptake of β-GLMMs in biomedicine remains limited, underscoring the need for increased methodological dissemination and software support. This gap is particularly relevant for immunological research, as antibody titer measurements represent a paradigmatic example of bounded longitudinal data. Antibody levels are indeed constrained by assay-specific limits, often show pronounced right skewness, and may accumulate at the upper detection limit following vaccination or booster doses; furthermore, repeated measurements within individuals induce correlation over time. These characteristics motivate the use of modeling approaches that explicitly respect bounded support and accommodate complex longitudinal dependence structures. To fill this gap, we aimed at demonstrating how β-GLMMs can be used to jointly characterize the mean antibody trajectory, its variability, and the probability of reaching the assay’s upper detection limit, leveraging SARS-CoV-2 antibody data from nursing home residents and healthcare workers from ten nursing homes (NH) in Calabria (southern Italy); moreover, we set ourselves the goal of identifying the socio-demographic and clinical factors associated with temporal evolution of antibody titers.

## 2. Materials and Methods

### 2.1. Study Design

A prospective longitudinal cohort study was conducted in five NHs in Calabria, Italy. NHRs and HCWs, in the context of their vaccination schedule, provided peripheral blood samples for serologic testing after second vaccine dose and then, regularly, every 28 days (within a window of ±14 days) for the first six months and every three months for the second six months (eight time points). All participants received two doses of BNT162b2 and a third booster (mRNA-1273 or BNT162b2) 6–9 months post-first dose.

The study began in February 2021, with written informed consent obtained from all study participants. This study was conducted in accordance with the Declaration of Helsinki. Eligibility criteria included age ≥18 years and at least one serologic test post-second dose.

**Figure 1:**
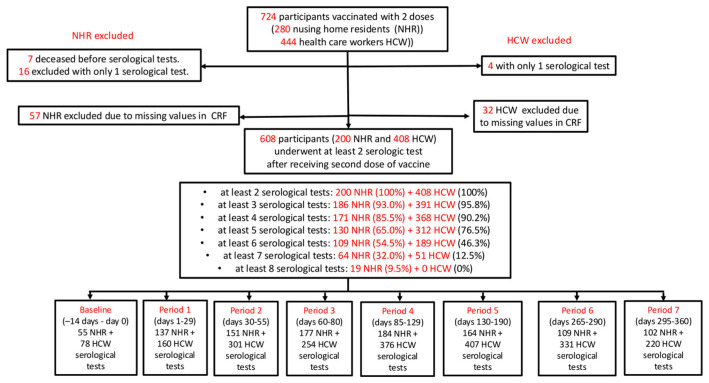
Recruitment of participants, testing, and follow-up of NHR and HCW samples.

Antibody levels were measured at baseline (two weeks pre-second dose, P0) and at eight time points every 4 weeks thereafter: P0 (−14–0 days), P1 (1–29 days), P2 (30–55 days), P3 (60–80 days), P4 (85–129 days), P5 (130–190 days), P6 (265–290 days), and P7 (295–360 days) (Figure 1).

Of 724 vaccinated individuals (280 NHRs and 444 HCWs), 116 were excluded due to incomplete data (4 HCWs and 16 NHRs because they had only one serologic assay result, 7 NHRs because they were deceased post-second dose and before undergoing serological tests; 32 HCWs and 57 NHRs because of missing values in clinical data); we then obtained a final sample of 608 participants (200 NHRs and 408 HCWs) for analysis.

Demographic and clinical data were collected at study enrolment and included age, gender, body mass index (BMI), and history of chronic diseases (with the latter detailed in Appendix A.1). The study design was approved by the Ethics Committee of the University of Calabria (protocol 006223, 15 March 2021).

### 2.2. SARS-CoV-2 IgG Immunoassays

Blood samples were properly prepared and stored following a standardized procedure. Blood samples were collected in Serum Separator Tubes (BD Diagnostic Systems, Franklin Lakes, NJ, USA).

Within 1 h from the collection, serum samples were immediately tested for the presence of SARS-CoV-2 antibodies using Elecsys Anti-SARS-CoV-2, an electrochemiluminescence immunoassay (sensitivity: 96.6%; specificity: 100%; sensitivity refers to sample >14 days after disease diagnosis), on a COBAS 601 platform (Roche, Basel, Switzerland), targeted on total Immunoglobulins (IgT) against S-protein RBD. The Roche Anti-SARS-CoV-2-S test has a signal range that spans from 0.4 to 250 U/mL that is further extended to 2500 U/mL thanks to a 1:10 dilution automatically performed by the instrument.

### 2.3. Statistical Analysis

Based on what was described in previous sections, after second vaccination, individuals were subjected, at fixed intervals, to serological tests. Let i = 1, …, N index individuals within each group (N = 200 for NHRs and 408 for HCWs), and let j = 0, …, 7 index the eight study periods (P0–P7). Formally, we denote by ${Titer}_{ij}$ the titer measurement for the i-*th* individual in the j-*th* interval. ${Titer}_{ij}$ can take continuous values in the limited range [0.4, 2500]; because antibody titers are bounded by the assay’s upper detection limit of 2500 U/mL, denoting with ${Tr}_{ij}=\frac{{Titer}_{ij}}{max\left\{{Titer}_{ij}\right\}}$, we defined a relative measure of antibody titer using the following transformation:

(1) $$Y_{ij}=1-{Tr}_{ij}=\frac{max\left\{{Titer}_{ij}\right\}-{Titer}_{ij}}{max\left\{{Titer}_{ij}\right\}}.$$

where $Y_{ij}$ can be interpreted as relative measure of absence of antibody titer (or the fraction of antibodies needed to reach the maximum response) and ranges between 0 (in case of ${Titer}_{ij}=max\left\{{Titer}_{ij}\right\}$, i.e., when in the j-*th* interval, for the i-*th* individual, a titer equal to the maximum measurable level is observed) and 0.99984 (i.e., when a titer equal to the minimum measurable titer is observed). So, for example, if $Y_{ij}$ = 0.2, we can say that 20% of antibodies are missing to reach the maximum measurable value (or the fraction of antibodies needed to reach the maximum response is 20%).

Preliminary inspection of the data revealed several features relevant for model choice. Antibody titers were bounded by the assay limits and showed pronounced right-skewness, with a substantial proportion of observations reaching the upper detection limit after vaccination or booster doses. Moreover, repeated measurements within individuals were correlated over time. These characteristics motivated the use of a modeling framework specifically designed for bounded longitudinal outcomes with within-subject dependence. Beta-GLMMs are the best fit as they to simultaneously estimate a non-linear model for the mean of $Y_{ij}$ for the probability that the i-*th* individual in the j-*th* interval has an antibody titer value equal to the maximum measurable value, i.e., $\mathrm{Pr}\left(Y_{ij}=0\right)=Pr\left({Tr}_{ij}=1\right)$ and for a dispersion indicator; furthermore, it allows for taking into account the longitudinal/clustered nature of the observed data. The methodology used is described in Appendix A.2.

### 2.4. Model Building Strategies

β-GLMMs were fitted using the glmmTMB package in R. The model jointly specifies three components: (i) a mean submodel describing the expected antibody trajectory over time, (ii) a precision submodel capturing changes in variability, and (iii) a submodel for the probability of observing values at the assay’s upper detection limit. Random intercepts were included at the individual level to account for within-subject correlation. Covariates were selected a priori based on clinical relevance. We started the analyses by estimating a “minimal” model for both NHR and HCW titers. This “minimal” model included the following covariates: gender (1 for males, 0 for females), age, day (days after the second dose vaccine), BMI (coded with three dummies variables to indicate the following classes: underweight with BMI ≤ 18.5 kg/m^2^, normal weight with 18.5 < BMI < 25 kg/m^2^, and lastly overweight with BMI ≥ 25 kg/m^2^), third dose (TD) (1 for subjects who received a third dose (booster) vaccine, 0 otherwise), and SARS-CoV-2 infection (C19) (1 for subjects who contracted SARS-CoV-2 infection before or during the study, 0 otherwise). In addition to this “minimal” model, more complex (extended) models were estimated, also including coexisting chronic diseases, which may impact the levels and kinetics of antibody titers. All variables reported in Table 1 with a prevalence greater than 5% were considered as candidate predictors. Variable selection and model selection were conducted using the Akaike Information Criterion (AIC) to evaluate and compare the performance of competing fitted models [9].

Statistical analyses were performed using R (version 4.3.1; R Foundation for Statistical Computing) using the following packages: betareg (3.2-1), glmmTMB (1.1.10), ggplot2 (3.5,1), and ggpubr (0.6.0).

## 3. Results

### 3.1. Study Population

Table 1 presents the demographic and health characteristics of the study participants.

Mean age was 81.3 for NHRs and 44.9 years for HCWs, respectively. Women were predominant in both groups (77.8% in NHRs, 53.2% in HCWs). As expected, NHRs had a higher burden of chronic diseases, particularly cardiorespiratory, neurodegenerative, and gastrointestinal diseases. The number of subjects with neutralizing antibody assays over time is shown in Figure 1. Overall, 1079 and 2127 serological tests were performed in NHRs and HCWs, respectively. Most participants had at least three serological assays, but only 9.5% of NHRs and no HCWs completed all eight assays.

### 3.2. Antibody Response over Time

Table 2 summarizes the fraction of antibodies needed to reach the maximum response, Y, across eight survey periods for both groups.

Several differences emerged between the two groups; in fact, in P0, both groups presented very high mean values of the fraction of antibodies necessary to reach the maximum response (0.9729 and 0.9831 for NHRs and HCWs, respectively); after having received the second dose in P1, the mean reduction in antibody levels was steeper for HCWs than NHRs (72% vs. 28%, respectively). After the third dose (in P7 for HCWs and in P6 and P7 for NHRs), a very strong reduction in mean levels was observed for both groups. The mean levels in NHRs were consistently higher than those of HCWs, suggesting a weaker immune response.

**Figure 2:**
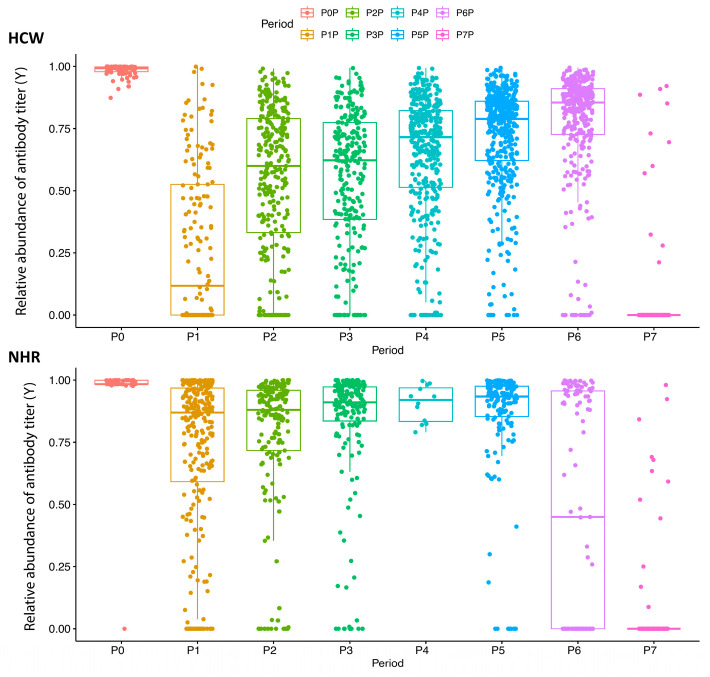
Distribution of antibodies 12 months after receipt of second dose of BNT162b2 vaccine. Vertical axis represents the fraction of antibodies required to reach the maximal response (Y), while the horizontal axis represents the eight survey periods (P0–P7) in HCWs (**upper** panel) and NHRs (**lower** panel).

Figure 2 illustrates boxplot diagrams of antibody levels over 12 months post-second dose. Because antibody titers were transformed, values close to zero indicate titers near the upper detection limit, whereas higher values indicate lower antibody levels. At P1, Y reached its lowest point for both groups: 0.118 in HCWs 0.87 in NHRs.

In HCWs, the median of Y follows a logarithmic increase until P7, where the third dose caused a sharp decline to 0. In NHRs, decline was slower and more linear, with a more gradual drop at P6 and P7. The peculiar evolution pattern of humoral immunity observed in NHRs in the last observational periods is, at least partially, related to the older age of NHRs which granted them the highest priority to receive a SARS-CoV-2 vaccine. Consequently, the response to the third booster dose of vaccine was detected first in NHRs and then in HCWs.

### 3.3. Beta-Generalized Linear Mixed Models

To clarify how cohort, demographic, immunological, and clinical data affected antibody response to vaccination, several β-GLMMs were fitted. As reported in Appendix A.2, these were fitted to simultaneously specify a regression model for the mean, μ, a regression model for the precision parameter, ϕ, and a regression model for α, i.e., the probability to reach the maximum response in terms of measurable antibody titer (corresponding to the maximum value of antibody titer).

### 3.4. “Minimal” Models for NHR and HCW Data

Table S1, reported in the Supplementary Files, presents parameter estimates for the minimal β-GLMMs with random intercepts on the mean and probability of maximum response. Table S2 displays predicted means and probabilities across the eight time periods. After the second dose in P1, the probability of reaching the maximum antibody response was 0.1439 in NHRs and 0.7469 in HCWs. After the third dose (P7 for HCWs, P6-P7 for NHRs), both groups showed increased probability, though such probability remained lower for NHRs.

Table S3 reports estimates from extended minimal models, including C19 and TD status. Tables S4 and S5 show estimated means and probability of maximum response under different combinations of C19 and TD status in NHRs and HCWs. Across groups, TD increased mean antibody levels and probability of peak response, especially in those with prior C19. These effects were stronger in HCWs than NHRs.

### 3.5. Effect of Covariates on Response

Table S5 shows minimal model estimates for the mean of $Y_{ij}$. In both groups, age, TD, and C19 significantly influenced antibody response. As exponentiated coefficients can be interpreted in terms of odds ratios, among NHRs with C19, the ratio between the expected fraction of antibodies needed to reach the maximum response, μ, and the expected fraction of antibodies observed (1 − μ) is about exp(−0.8015) = 0.4486 times higher than in subjects who did not experience infection. In other words, the fraction of antibodies needed to reach the maximum response is 2.23 times lower in NHRs with C19 than in NHRs who did not experience infection. Similarly, TD reduced Y by 8.05-fold. In HCWs, C19 and TD reduced Y by 7.09-fold and 1.95-fold, respectively. Regarding the effect of age, each 10-year age increase raised Y by 1.27 times in NHRs and 1.23 in HCWs. In NHRs, underweight and overweight BMI also correlated with higher Y (lower response), though BMI had no significant effect in HCWs.

Table S6 also presents estimates for the probability of reaching the maximum antibody response. C19 increased this probability by 11.3-fold in NHRs and 64-fold in HCWs; TD raised it by 397-fold in NHRs and 350-fold in HCWs. Age and underweight BMI negatively affected the probability in both groups. Finally, a quadratic relationship between the day variable and the probability of reaching maximum response was observed (Appendix A.3). All these fold-change estimates should be interpreted in the context of the underlying baseline risk, as large relative increases (as in the case of C19 and TD) may arise from low baseline probabilities.

### 3.6. Temporal Effects and U-Shaped Patterns

**Figure 3:**
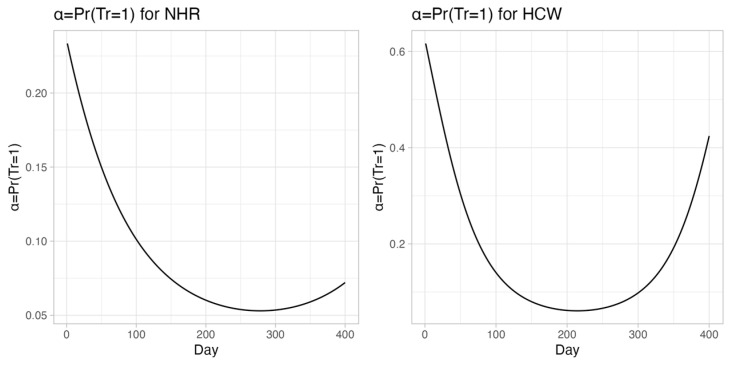
Probability of reaching the maximum antibody response after second vaccine dose in NHRs (**right**) and HCWs (**left**) based on parameter estimates reported in Table S6.

Figure 3 shows the predicted probability of achieving the peak response over time.

A U-shaped curve was observed in both groups: an initial post-vaccination increase, a decline over time, and recovery post-booster. The minimum value was estimated at day 278 for NHRs and 214 for HCWs. These minima align with the TD administration.

### 3.7. Extended Models for NHRs

Table 3 reports estimates from the extended β-GLMM for NHRs, incorporating comorbidities.

Conditions associated with lower antibody response (higher Y) included non-Alzheimer’s dementia, atrial fibrillation, COPD, and underweight BMI, along with age and day. In contrast, prior C19, TD, hypertension, and stroke were associated with stronger responses.

In terms of maximum response probability, positive predictors included female sex, non-Alzheimer’s dementia, prior C19, and TD. Age, PAD, HF, and underweight BMI were negative predictors.

Several covariates also influenced the precision of Y. Age, female sex, underweight BMI, day, HF, and non-Alzheimer’s dementia were linked to higher precision (lower variability); conversely, C19, TD, and stroke were associated with increased variability in the humoral response.

## 4. Discussion

### 4.1. Antibody Kinetics Following Vaccination in NHRs and HCWs

Antibody titers are a key marker to evaluate vaccine-induced immunity protection. In the context of the anti-SARS-CoV-2 vaccination campaign, our findings highlight the relationship between vaccination timing and antibody response kinetics in subjects of different ages; antibody titers peaked four weeks post-vaccination in both NHRs and HCWs and then declined until they received the third booster dose; while HCWs showed a rapid exponential antibody decrease, NHRs experienced a slower and linear decline. These results confirm what was reported by previous studies [21,22,23,24] and highlight the necessity of booster doses, particularly for frail subjects. NHRs also exhibited weaker and more variable responses, likely influenced by aging-related immunosenescence [25,26,27,28].

### 4.2. Impact of Prior Infection and Booster Doses on Immune Response

Our results demonstrate a strong correlation between SARS-CoV-2 infection, vaccination, and antibody levels. Natural infection can induce production of proinflammatory cytokines [23] and amplify vaccine response through “hybrid immunity” [26,29]. Consistent with previous studies [29,30,31], NHRs with past SARS-CoV-2 infection had enhanced immune responses, though to a lesser extent than HCWs. In contrast to previous studies [21,31,32], our investigation uniquely assessed not only median IgG levels but also the probability of reaching a peak response and antibody variability. Interestingly, both booster doses and prior infection significantly increased the probability to reach the peak antibody response, but the effect was stronger in HCWs (64 times) compared to NHRs (11 times, compared to noninfected individuals) (Table S6). Post-second dose antibody decline was counteracted by booster dose, eliminating the immune response gap between NHRs and HCWs.

### 4.3. Role of Comorbidities and Clinical Characteristics

Several clinical factors influenced immune response in NHRs; indeed, age, BMI ≤ 18.5 kg/m^2^, AF, non-Alzheimer’s dementia, and COPD correlated with lower antibody levels, whereas C19, TD, hypertension, and stroke correlated with higher responses. Age, PAD, and HF were associated with a lower probability of reaching peak response, while C19, TD, female sex, and non-Alzheimer’s dementia increased it.

The role of BMI and comorbidities in vaccine responses was underexplored among NHRs; Meyers E. et al. showed that cardiometabolic diseases, nephrological comorbidities, and cancer may weaken immune response [33]; hyperpolypharmacy was also linked to weaker responses [34]. Our findings contribute to a better understanding of the complex interactions between chronic diseases and immune response; interestingly, among chronic diseases, only hypertension and stroke were associated with stronger immune responses, in contrast with previous studies reporting a negative relationship [35,36,37]. Indeed, Watanabe et al. found that hypertension was associated with lower antibody titers following COVID-19 vaccination, likely due to chronic inflammation and endothelial damage [35]; similarly, Soegiarto et al. reported that hypertensive individuals had a higher risk of breakthrough infections after vaccination, suggesting impaired vaccine efficacy [36]; a recent study revealed that hypertensive older patients with higher frailty levels experienced a more pronounced antibody reduction compared to patients with lower frailty [37]. The discrepancies between our study and previous ones may stem from differences in study populations, with frail and older adults often underrepresented in previous studies, as well as from the complex interplay between duration of hypertension and immune function.

Hypertension is characterized by chronic inflammation, endothelial dysfunction, and immune activation, which could influence vaccine-induced immunity. While chronic inflammation and endothelial dysfunction are thought to potentially impair immune responses and vaccine efficacy, long-lasting hypertension may induce sustained activation of both the adaptive and innate immune systems [38]; this immune activation, driven by pro-inflammatory pathways, may boost vaccine response. Additionally, renin–angiotensin system (RAS) dysregulation might contribute to enhanced post-vaccination response; the binding of circulating spike proteins from the vaccine to angiotensin-converting enzyme 2 (ACE2) can affect the balance between angiotensin II and its inactive form, angiotensin 1–7, which normally has a protective, hypotensive effect [39,40]. This inflammatory response may be particularly pronounced in individuals with pre-existing hypertension, as their immune systems may react more vigorously to the vaccine, potentially leading to elevated blood pressure and hyperinflammatory reactions. Further research is needed to clarify the mechanisms underlying this association and to determine whether specific antihypertensive medications modulate vaccine responses. Along with hypertension, stroke was also associated with enhanced immune response in NHRs, potentially due to increased activation of innate and acquired immunity following cerebrovascular events; stroke disrupts local and systemic immunity [41], causing long-term dysregulation, which may lead to enhanced and cross-reactive immune response following vaccination; additionally, stroke-induced immunological perturbations could increase latent SARS-CoV-2 infections [42], further amplifying IgG titers; however, this finding warrants further investigation to determine the underlying mechanisms involved.

### 4.4. Nutritional Status and Frailty-Related Immune Modulation

Undernutrition, common among NHRs, has been associated with decreased vaccine efficacy [43,44] due to deficiencies in essential vitamins and minerals necessary for robust immune function [44,45], thus reducing the ability to mount an effective response to vaccination. Our findings underscore the importance of nutritional status as part of comprehensive vaccination strategies for frail subjects.

Cardiovascular disease’s negative impact on vaccine response has been reported in previous studies; indeed, a higher prevalence of cardiovascular comorbidities was found among seronegative compared with seropositive individuals after vaccination [46]; furthermore, cardiovascular medications may modulate immune response and potentially reduce IgG titers in vaccinated individuals [47]; a previous study found that men with cardiovascular diseases had a weaker humoral response post-vaccination against SARS-CoV-2, aligning with our results [46].

Non-Alzheimer’s dementia was also associated with a decreased immune response, consistently with evidence that neurodegenerative diseases increase the prevalence and severity of COVID-19 as well as the risk of breakthrough infections [48,49,50,51]; immune dysregulation linked to neurodegeneration may underlie reduced vaccine efficacy [52,53]. However, Alzheimer’s dementia was not linked to antibody titers in our cohort, suggesting variations in immune function between dementia types. In this regard, Alzheimer’s disease is characterized by neuroinflammatory pathways that may not directly impair vaccine-induced immunity, whereas other forms of dementia may have more pronounced effects on immune function. Indeed, B and T cells tend to decrease in all forms of dementia, and monocytes and NK cells are decreased only in vascular dementias but not in Alzheimer’s disease [52]. These cells were shown to be particularly relevant for mounting effective response following SARS-CoV-2 vaccination [50,53,54].

The findings from our study have important implications for vaccination strategies in frail populations, particularly NHRs. Given the associations between comorbidity, nutritional status, and vaccine response, personalized vaccination strategies should be considered. Undernourished NHRs may benefit from targeted nutritional support, while those with cardiovascular diseases or dementia may require tailored vaccination schedules. Enhanced immune responses in hypertensive or stroke-affected NHRs suggest that these individuals may gain greater protection from booster doses. Future studies should explore the biological mechanisms underpinning these associations, incorporating both humoral and cellular immune assessments.

### 4.5. Methodological Considerations

A major strength of this study is the use of a robust statistical model (beta-generalized linear mixed model) to evaluate multiple parameters influencing immune response and allowing for the simultaneous specification of a regression model for the mean, μ, of the humoral response, a second one for the precision parameter, ϕ, and a third one for the probability to obtain the maximum immune response, α. This approach allowed us to identify with greater accuracy the key demographic and clinical characteristics affecting antibody response.

Our real-world data provide valuable insights for shaping ongoing and future vaccination campaigns. The distinct immune response trajectories observed between NHRs and HCWs reinforce the need for timely booster vaccinations, particularly for frail older populations who exhibit suboptimal immune response.

While our findings offer critical insights, some limitations should be acknowledged. Firstly, the relatively small sample size may limit generalizability. Additionally, our study focused solely on humoral immunity, omitting cellular and cytokine responses. On the other hand, our use of a single vaccine type (BNT162b2) and centralized laboratory testing enhance the reproducibility and reliability of study results. Moreover, humoral response remains a crucial component for clearance of cytopathic viruses and reinfection prevention, and its standard measurement facilitates comparison across studies.

## 5. Conclusions

This study provides a comprehensive analysis of antibody responses to BNT162b2 vaccination in older NHRs and younger HCWs in southern Italy. Our findings highlight distinct immune trajectories between these populations, with frail older NHRs showing delayed and attenuated responses that are partially restored by booster doses. Importantly, prior SARS-CoV-2 infection and booster vaccination substantially increased the probability of achieving peak antibody responses, underscoring the relevance of hybrid immunity and repeated antigen exposure. Clinical factors such as comorbidities, nutritional status, and age significantly influenced vaccine responsiveness, emphasizing the need for personalized vaccination strategies in frail and institutionalized populations. The observed associations between certain comorbidities and enhanced immune responses further suggest complex immunological mechanisms that warrant future investigation.

## Figures and Tables

**Table 1:** Descriptive statistics of characteristics of study participants in NHR and HCW groups. (SD: standard deviation).

Parameter	NHRs (N = 200)	HCWs (N = 408)
Age, mean (SD)	82.3 (10.8)	44.9 (10.4)
Male sex, *n* (%)	44 (22.2)	191 (46.8)
Height (m), mean (SD)	1.60 (0.08)	1.69 (0.09)
Weight (kg), mean (SD)	62.4 (13.1)	73.0 (15.2)
BMI (kg/m^2^), mean (SD)	24.3 (4.7)	25.5 (4.2)
Hypertension, *n* (%)	137 (68.5)	70 (17.2)
DVT/Pulmonary embolism, *n* (%)	8 (4.0)	3 (0.7)
CAD, *n* (%)	72 (36.0)	8 (2.0)
AF, *n* (%)	33 (16.5)	5 (1.2)
Orthostatic hypotension, *n* (%)	3 (1.5)	11 (2.7)
PAD, *n* (%)	67 (33.5)	12 (2.9)
HF, *n* (%)	19 (9.5)	0 (0)
TIA, *n* (%)	72 (36)	8 (1.9)
Stroke, *n* (%)	32 (16)	2 (0.5)
Hemiparesis/Hemiplegia, *n* (%)	13 (6.5)	1 (0.2)
Alzheimer’s dementia, *n* (%)	63 (31.5)	0 (0)
Non-Alzheimer’s dementia, *n* (%)	55 (27.5)	0 (0)
Parkinson’s disease, *n* (%)	12 (6)	0 (0)
Epilepsy, *n* (%)	6 (3.0)	0 (0)
Gastritis, *n* (%)	42 (21.0)	53 (13)
Peptic ulcer, *n* (%)	8 (4.0)	5 (1.2)
Dyspepsia, *n* (%)	48 (24)	24 (5.9)
Constipation, *n* (%)	42 (21)	30 (7.4)
Diarrhea, *n* (%)	14 (7)	3 (0.7)
Diverticular disease, *n* (%)	6 (3)	5 (1.225)
Type 2 diabetes mellitus, *n* (%)	52 (26)	15 (3.7)
Clinical hyperthyroidism, *n* (%)	5 (2.5)	3 (0.7)
Subclinical hyperthyroidism, *n* (%)	5 (2.5)	3 (0.7)
Clinical hypothyroidism, *n* (%)	10 (5)	26 (6.4)
Subclinical hypothyroidism, *n* (%)	7 (3.5)	6 (1.5)
Dyslipidemias, *n* (%)	42 (21)	38 (9.3)
Arthrosis, *n* (%)	107 (53.5)	22 (5.4)
Rheumatoid arthritis, *n* (%)	1 (0.5)	12 (2.9)
Osteoporosis, *n* (%)	99 (40.5)	12 (2.9)
Asthma, *n* (%)	3 (1.5)	9 (2.2)
Emphysema, *n* (%)	3 (1.5)	1 (0.2)
COPD, *n* (%)	82 (41.0)	6 (1.5)
Glaucoma, *n* (%)	4 (2.0)	3 (0.7)
Deafness, *n* (%)	20 (10.0)	0 (0)
Macular degeneration, *n* (%)	12 (6.0)	2 (0.5)
Cataract, *n* (%)	19 (9.5)	4 (1)
Celiac disease, *n* (%)	1 (0.5)	4 (1)
Coagulation disorders, *n* (%)	6 (3)	7 (1.7)
Carotid atheromasia, *n* (%)	47 (23.5)	0 (0)
Alternating bowel habits, *n* (%)	19 (9.5)	20 (4.9)

Notes: AF = atrial fibrillation; BMI = body mass index; CAD = coronary artery disease; COPD = chronic obstructive pulmonary disease; DVT = deep vein thrombosis; HF = heart failure; PAD = peripheral artery disease; TIA = transient ischemic attack.

**Table 2:** Descriptive statistics of the fraction of antibodies needed to reach the maximum response in NHRs and HCWs.

	Index/Period	P0	P1	P2	P3	P4	P5	P6	P7
NHRs	Number of tests per period	55	137	151	177	184	164	109	102
	Mean	0.9729	0.701	0.724	0.769	0.826	0.8543	0.4709	0.067
	Median	0.984	0.882	0.85	0.88	0.913	0.9328	0.4496	0
	Variance	0.0176	0.128	0.028	0.085	0.066	0.0525	0.2083	0.062
	Standard deviation	0.1326	0.358	0.167	0.291	0.257	0.2292	0.4565	0.25
	Coefficient of variation	0.1363	0.511	0.231	0.379	0.31	0.2682	0.9692	3.738
HCWs	Number of tests per period	78	160	301	254	376	407	331	220
	Mean	0.9831	0.275	0.539	0.546	0.638	0.7125	0.7642	0.032
	Median	0.9922	0.118	0.6	0.622	0.716	0.7888	0.8548	0
	Variance	0.0005	0.097	0.09	0.08	0.064	0.0494	0.0593	0.022
	Standard deviation	0.0233	0.312	0.3	0.283	0.254	0.2222	0.2434	0.149
	Coefficient of variation	0.0237	1.133	0.556	0.518	0.397	0.3119	0.3186	4.702

HCWs: healthcare workers; NHRs: nursing home residents.

**Table 3:** Estimates of the final model for the mean with random intercepts, for the probability of reaching maximum antibody response, and for the precision parameter for the NHRs group.

**Conditional Model**		
**Parameter**	**Estimate**	***p*-Value**
C19	−0.824	2.27 × 10^−7^
TD	−2.577	1.79 × 10^−11^
Age	0.022	<2.00 × 10^−16^
Day^2^	9.25 × 10^−6^	0.014
BMI < 18.5	0.381	0.004
BMI > 25	0.099	0.184
Hypertension	−0.261	0.001
Non-Alzheimer’s dementia	0.289	0.002
AF	0.441	4.02 × 10^−5^
Stroke	−0.212	0.065
Alternating bowel habits	0.183	0.156
COPD	0.120	0.086
**Zero-Inflaction Model**		
	**Estimate**	***p*-Value**
C19	2.259	5.52 × 10^−13^
TD	6.093	3.91 × 10^−16^
Age	−0.029	<2.00 × 10^−16^
Gender	0.593	0.017
Day	−0.013	0.002
Day^2^	2.505	0.089
PAD	−0.885	0.002
Non-Alzheimer’s dementia	0.711	0.007
HF	−0.892	0.051
**Dispersion Model**		
	**Estimate**	***p*-Value**
C19	−0.693	1.31 × 10^−4^
TD	−1.983	8.65 × 10^−10^
Age	0.008	1.26 × 10^−8^
Gender	0.178	0.054
BMI < 18.5	1.101	1.97 × 10^−8^
Day	0.006	<2.00 × 10^−16^
HF	0.264	0.083
Non-Alzheimer’s dementia	0.446	2.51 × 10^−4^
AF	0.616	5.82 × 10^−5^
Stroke	−0.287	0.033
PAD	0.146	0.110
AIC	−1457.9	

Notes: AIC = Akaike Information Criterion; BMI = body mass index; C19 = SARS-CoV-2 infection; HF = heart failure; NHRs: nursing home residents; PAD = peripheral artery disease; TD = third dose.

## Data Availability

Data will be available on reasonable request to the corresponding author.

## Supplementary Materials

The following supporting information can be downloaded at: https://www.mdpi.com/article/10.3390/v18010109/s1; Table S1: β-GLMM with random intercepts, both on the mean and on the probability of reaching the maximum antibody response, under a homogeneity hypothesis for both groups. Table S2: Estimates of the means and probabilities of reaching the maximum response across the eight periods for the two groups. Table S3: Estimates of the regression models with random intercepts for the mean and for the probability of reaching the maximum antibody response and for the precision parameter. Table S4: Estimates of the means and probabilities of reaching the maximum antibody response in NHRs. Table S5: Estimates of the means and probabilities of reaching the maximum antibody response in HCWs. Table S6: Estimates of the “minimal” regression models for the mean with random intercepts, for the probability of reaching the maximum antibody response, and for the precision parameter for the NHR group.

## Abbreviations

The following abbreviations are used in this manuscript:

NHRs	Nursing Home Residents
COVID-19	Coronavirus Disease 2019
HCWs	Healthcare Workers
SARS-CoV-2	Severe Acute Respiratory Syndrome-related Coronavirus 2
BMI	Body Mass Index
IgG	Immunoglobulin G
NH	Nursing Home
mRNA	Messenger Ribonucleic Acid
COPD	Chronic Obstructive Pulmonary Disease
OECD	Organisation for Economic Co-operation and Development
RBD	Receptor-Binding Domain
PCR	Polymerase Chain Reaction
CAD	Coronary Artery Disease
DVT	Deep Vein Thrombosis
PAD	Peripheral Artery Disease
HF	Heart Failure
TIA	Transient Ischemic Attack
ECLIA	Electrochemiluminescence Immunoassay
IgT	Immunoglobulin Total
TD	Third Dose
C19	COVID-19 infection
β-GLMMs	Beta-Generalized Linear Mixed Models
AIC	Akaike Information Criterion
AF	Atrial Fibrillation
RAS	Renin–Angiotensin System
ACE2	Angiotensin-Converting Enzyme 2

## Appendix A

### Appendix A.1

History of chronic diseases included the following:

(1) Cardiovascular diseases: hypertension, coronary artery disease (CAD), carotid vasculopathy, pulmonary embolism, atrial fibrillation, deep vein thrombosis (DVT), orthostatic hypotension, peripheral artery disease (PAD), heart failure (HF);
(2) Cerebrovascular diseases: transient ischemic attack (TIA), stroke, Alzheimer’s and non-Alzheimer’s dementias, Parkinson’s disease, epilepsy, hemiparesis/hemiplegia;
(3) Gastrointestinal diseases and symptoms: gastritis, peptic ulcer, dyspepsia, constipation, diarrhea, diverticular diseases, alternating bowel habits;
(4) Metabolic and endocrine diseases: type 2 diabetes mellitus, clinical and subclinical hypo- and hyperthyroidism, Hashimoto’s thyroiditis, dyslipidaemia;
(5) Joint and bone diseases: arthrosis, rheumatoid arthritis, osteoporosis;
(6) Respiratory diseases: chronic obstructive pulmonary disease (COPD), asthma, emphysema;
(7) Diseases of sensory systems: glaucoma, macular degeneration, cataract, deafness;
(8) Autoimmune diseases: systemic lupus erythematosus, celiac diseases, coagulation disorders;
(9) Previous severe allergic reactions.

### Appendix A.2

In the literature [55], in the case in which the observations are continuous and limited in the interval (0, 1), the use of a normal distribution is not appropriate, as it would attribute non-zero density to values that do not belong to the interval (0, 1). In such situations, the correct specification involves using the beta density function.

In this paper, we use an appropriate parametrization of the usual beta distribution, proposed by Ferrari and Cribari-Neto [56], which allows for a more immediate specification of the regression and simple interpretation of the regression coefficients. Denoted by Y for a continuous random variable in the interval (0, 1), the reparametrized beta density function is:

(A1) $$f\left(y;\mu ,\phi \right)=\frac{1}{B\left(\mu \phi ,\;\;\left(1-\mu \right)\phi \right)}\;y^{\mu \phi -1}{\left(1-y\right)}^{\left(1-\mu \right)\phi -1}\;$$

where y∈(0,1), μ∈(0,1), ϕ>0, and B(.,.) is the mathematical beta function, defined by $B\left(\mu \phi ,\;\left(1-\mu \right)\phi \right)=\int_{0}^{1}y^{\mu \phi -1}{\left(1-y\right)}^{\left(1-\mu \right)\phi -1}dy$.

The parameter μ denotes the expected value of Y, i.e., $E\left(Y\right)=\mu$. Since the variance of Y is given by $V\left(Y\right)=\frac{\mu (1-\mu )}{\phi +1}$, the parameter ϕ is interpreted as a precision parameter, in the sense that, for fixed values of μ, the greater the value of ϕ, the smaller the variance of Y.

To account for the possibility that the relative measurement $Y_{ij}$ may take values equal to zero, it is necessary to use a mixture of two distributions: a beta distribution for y∈(0,1) and a degenerate distribution at zero. In this case, the probability density function of the variable Y is given by:

(A2) $$f\left(y;\mu ,\phi ,\;\alpha \right)=\left\{\begin{array}{c} \alpha \;\;\;\;\;\;\;\;\;\;\;\;\;\;\;\;\;\;\;\;\;\;\;\;\;\;\;\;\;\;\;\;\;if\;\;y=0\\ \left(1-\alpha \right)f\left(y;\mu ,\phi \right)\;\;\;\;\;\;if\;y\in (0,1) \end{array}\right.$$

where α is the probability of observing zero, i.e., α=Pr(Y=0). In the literature, the density described by (A2) is called the zero-inflated beta density [57].

With reference to the measurement of the absence of antibody titers ($Y_{ij}$), it is important to note that $\alpha =\mathrm{Pr}\left(Y_{ij}=0\right)=Pr\left({Tr}_{ij}=1\right)$, i.e., α coincides with the probability that the *i-th* individual in the *j-th* interval has an antibody titer value equal to the maximum measurable value. Note that *i* ranges between 1 and *N* (with *N* = 200 for NHRs and 408 for HCWs); *j* ranges between 0 and 7 (corresponding to the 8 analyzed periods).

The beta regression model, using the construction logic of generalized linear models, provides the possibility of simultaneously specifying a regression model for the mean, μ, a regression model for the precision parameter, ϕ, and a regression model for α, i.e., for the probability that Y = 0, as follows:

Let $y_{1},\;y_{2},\;\ldots ,\;y_{n}$ be independent random variables such that each $y_{i}$ has a probability density function (A2) with $\mu =\mu_{i}$, $\phi =\phi_{i}$, and $\alpha =\alpha_{i}$ for *i* = 1, …, *n*. Moreover, for each individual *i*, we assume that we observe the following vectors of covariates: $x_{1,i.}^{\prime }=\left(1,x_{1,i1},\;x_{1,i2},\;\ldots ,\;x_{1,ik_{1}}\right)$, $x_{2,i.}^{\prime }=\left(1,x_{2,i1},\;x_{2,i2},\;\ldots ,\;x_{2,ik_{2}}\right)$ and $x_{3,i.}^{\prime }=\left({1,x}_{3,i1},\;x_{3,i2},\;\ldots ,\;x_{3,ik_{3}}\right)$. Using appropriate link functions, we can write the following:

(A3) $$\left\{\begin{array}{c} h_{1}\left(\mu_{i}\right)=x_{1,i.}^{\prime }\beta_{1}\\ h_{2}\left(\phi_{i}\right)=x_{2,i.}^{\prime }\beta_{2}\\ h_{3}\left(\alpha_{i}\right)=x_{3,i.}^{\prime }\beta_{3} \end{array}\right.$$

where $\beta_{1}^{\prime }=\left(\beta_{10},\beta_{11},\;\beta_{12},\;\ldots ,\;\beta_{1k_{1}}\right)$, $\beta_{2}^{\prime }=\left(\beta_{20},\beta_{21},\;\beta_{22},\;\ldots ,\;\beta_{2k_{2}}\right)$, and $\beta_{3}^{\prime }=\left(\beta_{30},\beta_{31},\;\beta_{32},\;\ldots ,\;\beta_{3k_{3}}\right)$ are vectors of unknown regression parameters. In the literature, the specifications (A2) and (A3) are called zero-inflated beta regression [57]. To take into account the variation range of the parameters $\mu_{i},\;\phi_{i}\;and\;\alpha_{i}$, we chose the following link functions:

(A4) $$\left\{\begin{array}{c} logit\left(\mu_{i}\right)=log\left(\frac{\mu_{i}}{1-\mu_{i}}\right)=x_{1,i.}^{\prime }\beta_{1}\\ \;\;\;\;\;\;\;\;\;\;\;\;\;\;\;\;\;\;\;\;\;\;\;\;\;\;\;\;log\left(\phi_{i}\right)=x_{2,i.}^{\prime }\beta_{2}\\ logit\left(\alpha_{i}\right)=log\left(\frac{\alpha_{i}}{1-\alpha_{i}}\right)=x_{3,i.}^{\prime }\beta_{3} \end{array}\right.\;\;\;\Rightarrow \left\{\begin{array}{c} \mu_{i}=\frac{e^{x_{1,i.}^{\prime }\beta_{1}}}{1+e^{x_{1,i.}^{\prime }\beta_{1}}}\\ \phi_{i}=e^{x_{2,i.}^{\prime }\beta_{2}}\\ \alpha_{i}=\frac{e^{x_{3,i.}^{\prime }\beta_{3}}}{1+e^{x_{3,i.}^{\prime }\beta_{3}}} \end{array}\right.$$

The *logit-link* ensures that for each value of the linear predictor, $\mu_{i}$ and $\alpha_{i}$ always fall within the interval (0, 1); similarly, the *log-link* ensures that for each value of the linear predictor, $\phi_{i}$ always takes values greater than zero.

Finally, to consider the longitudinal/clustered nature of the observed data, the literature [19] suggests adding a random component, called *random effects*, into the linear predictor for the mean in the following way:

(A5) $$logit\left(\mu_{ij}\right)=log\left(\frac{\mu_{ij}}{1-\mu_{ij}}\right)=x_{1,ij.}^{\prime }\beta_{1}+z_{ij.}^{\prime }b_{j}$$

where $b_{j}$ is the vector of random effects, assumed to be normally distributed, i.e., $b_{j}∼N(0,G)$. The specification reported in (A4) integrated with Equation (A5) is called the beta-generalized linear mixed model (Beta-GLMM).

In this paper, we only consider random intercepts to account for the time intervals of observation of the antibody titer; therefore, we set $z_{ij.}^{\prime }=1$.

β-GLMMs were fitted using the glmmTMB (1.1.10) package in R (see CRAN: package glmmTMB, https://cran.r-project.org/web/packages/glmmTMB/index.html, accessed on 30 July 2025). In particular, below, we report the standard instruction used to estimate the different models:

$$\begin{array}{c} model<-glmmTMB(y\sim x_{1,ij.}^{\prime }\beta_{1}+\left(1|Nperiod\right)-1,\\ ziformula=\sim \;x_{3,i.}^{\prime }\beta_{3}-1,\\ data=dataset,\\ disp=\sim \;x_{2,i.}^{\prime }\beta_{2},\\ \mathrm{control}=\mathrm{glmmTMBControl}(\mathrm{optimizer}=\mathrm{optim},\;\mathrm{optArgs}=\mathrm{list}(\mathrm{method}=“\mathrm{BFGS}”)),\\ family=beta\_family(link=“logit”)\;), \end{array}$$

where there are the following:

(i) A mean submodel describing the expected antibody trajectory over time ($y\sim x_{1,ij.}^{\prime }\beta_{1}+\left(1|Nperiod\right)-1)$;
(ii) A precision submodel capturing changes in variability ($disp\;=\;\sim \;x_{2,i.}^{\prime }\beta_{2})$;
(iii) A submodel for the probability of observing values at the assay’s upper detection limit ($ziformula\;=\;\sim \;x_{3,i.}^{\prime }\beta_{3}-1).$

Model parameters are estimated by maximizing the marginal likelihood, which is obtained by integrating out the unobserved random effects from the likelihood function [19]. The Akaike Information Criterion (AIC) was used to evaluate and compare the performance of the different fitted models [19].

### Appendix A.3

From Table S6, a particularly interesting aspect emerges regarding the link between the probability of reaching the maximum response and the *Day* variable, i.e., $\alpha =Pr\left({Tr}_{ij}=1\;|\;Day\right)$. For both groups, we estimate a quadratic relationship with the presence of a minimum in the observation period. In particular, for NHRs, this relationship is given by:

$$\alpha_{NHR}=Pr\left({Tr}_{ij}=1\;|\;Day\right)=\frac{e^{-1.177-0.01227\times Day+0.00002206\times {Day}^{2}}}{1+e^{-1.177-0.01227\times Day+0.00002206\times {Day}^{2}}}$$

with a minimum point given by $min\left\{Day\right\}=\frac{0.01227}{2\times 0.00002206}=278.1052$ days; meanwhile, for HCWs, we have:

$$\alpha_{HCW}=Pr\left({Tr}_{ij}=1\;|\;Day\right)=\frac{e^{0.5047-0.03022\times Day+0.00007049{Day}^{2}}}{1+e^{0.5047-0.03022\times Day+0.00007049{Day}^{2}}}$$

with a minimum point given by $min\left\{Day\right\}=\frac{0.03022}{2\times 0.00007049}=214.357\;$ days.
