# Supplementary material for: Statistical Modeling of Humoral Immune Response Dynamics to mRNA COVID-19 Vaccines in Nursing Home Residents and Healthcare Workers from Southern Italy
Source: Viruses. 2026 Jan 14;18(1):109. doi: 10.3390/v18010109 (PMC12846483; doi:10.3390/v18010109)
Supplement: Supplementary file 1 [file viruses-18-00109-s001.zip › viruses-4069310-supplementary.pdf]

Table S1. Beta GLMM with random intercepts both on the mean and on the probability of reaching the maximum antibody response, under homogeneity hypothesis for both groups.

		**NHRs**				**HCWs**			
		Estimate	Standard error	z value	p-value	Estimate	Standard error	z value	p-value
**Conditional model**									
	Intercept	1.6181	0.1886	8.581	$<2\times10^{-16}$	0.859	0.2631	3.264	0.0011
	Random Intercepts	P0	P1	P2	P3	P0	P1	P2	P3
		0.9305	0.1172	-0.2356	-0.1402	1.7044	-0.8544	-0.3934	-0.4266
		P4	P5	P6	P7	P4	P5	P6	P7
		0.0444	0.1123	0.1197	-0.9869	-0.192	0.0332	0.3686	-0.2609
**Zero-Inflaction model**									
	Intercept	-1.8031	0.6386	-2.824	0.00475	-2.0588	0.8728	-2.359	0.0183
	Random Intercepts	P0	P1	P2	P3	P0	P1	P2	P3
		-1.7316	0.0359	0.3922	-0.6358	-2.9545	1.8248	0.1532	-0.0698
		P4	P5	P6	P7	P4	P5	P6	P7
		-1.1229	-1.1208	1.6182	3.7095	-0.8129	-1.5944	-0.8483	4.9239
	**Dispersion parameter**	3.16				4.06			

Notes: NHR: Nursing Home Residents; HCW: Health Care Workers.

Table S2. Estimates of the means and probabilities of reaching the maximum response across the eight periods for the two groups.

NHRs						
	b_j	$log\frac{\mu_{ij}}{1-\mu_{ij}} = \beta + \beta_j$	$\mu_{ij} = \frac{e^{\beta+\beta_j}}{1+e^{\beta+\beta_j}}$	γ_j	$log\frac{P(Y_{ij}=0)}{1-P(Y_{ij}=0)} = \gamma + \gamma_j$	$P(Y_{ij}=0) = \frac{e^{\gamma+\gamma_j}}{1+e^{\gamma+\gamma_j}}$
P0	0.9305	2.5481	0.9274	-1.7315	-3.5331	0.0284
P1	0.1171	1.7281	0.8492	0.0359	-1.7672	0.1459
P2	-0.2356	1.3881	0.8003	-0.3922	-2.1913	0.1005
P3	-0.1402	1.4781	0.8143	-0.6358	-2.4331	0.0807
P4	0.0444	1.6621	0.8351	-1.1229	-2.926	0.0509
P5	0.1123	1.7301	0.8494	-1.1208	-2.9239	0.0510
P6	0.1197	1.7371	0.8503	1.6182	-0.1931	0.4519
P7	-0.9869	0.6312	0.6528	3.709	1.8969	0.8695
**HCWs**						
P0	1.7044	2.5634	0.9285	-2.9445	-5.0133	0.0059
P1	-0.8544	0.0046	0.5011	1.8248	-0.234	0.7489
P2	-0.3934	0.4656	0.6143	-0.1532	-1.9056	0.1449
P3	-0.4266	0.4324	0.6064	-0.0698	-2.1286	0.1128
P4	-0.1919	0.667	0.6608	-0.8129	-2.8717	0.0030
P5	0.0332	0.8922	0.7093	-1.5945	-3.6532	0.0129
P6	0.3686	1.2276	0.7734	-0.8483	-2.9071	0.0273
P7	-0.2609	0.5981	0.6452	4.9239	2.8651	0.9461

HCWs: Health Care Workers; NHRs: Nursing Home Residents.

Table S3. Estimates of the regression models with random intercepts for the mean and for the probability of reaching the maximum antibody response, and for the precision parameter.

	**NHRs**				**HCWs**			
	Conditional model							
	**Estimate**	**SE**	**z-value**	**p-value**	**Estimate**	**SE**	**z-value**	**p-value**
Intercept	1.8449	0.1329	13.877	$<2\times10^{-16}$	0.9321	0.2675	3.485	0.000492
C19	-0.7006	0.1599	-4.381	1.18×10^{-5}	-1.8241	0.2696	-6.767	1.32×10^{-11}
TD	-1.4133	0.3374	-4.189	2.81×10^{-5}	-0.5337	0.3294	-1.62	0.1052
Random intercepts	P0=0.7245<br>P1=-0.0409<br>P2=-0.3635<br>P3=-0.2664<br>P4=-0.0855<br>P5=-0.0156<br>P6=0.1038<br>P7=-0.0816				P0=1.6583<br>P1=-0.927<br>P2=-0.4622<br>P3=-0.4957<br>P4=-0.259<br>P5=-0.019<br>P6=0.352<br>P7=0.1369			
	Zero-inflaction model							
	**Estimate**	**SE**	**z-value**	**p-value**	**Estimate**	**SE**	**z-value**	**p-value**
Intercept	-3.0564	0.2516	-12.15	$<2\times10^{-16}$	-2.7606	0.5687	-4.854	1.21×10^{-6}
C19	2.173	0.2921	7.44	1.0×10^{-13}	4. 3071	0.4399	9.791	$<2\times10^{-16}$
TD	5.152	0.4667	11.04	$<2\times10^{-16}$	4.2206	0.6737	6.265	3.73×10^{-10}
Random intercepts	P0=-0.2022<br>P1=0.7492<br>P2=0.3736<br>P3=0.0673<br>P4=-0.2771<br>P5=-0.2851<br>P6=-0.2438<br>P7=-0.1077	P1	P2	P3	P0=-1.812<br>P1=2.4209<br>P2=0.8448<br>P3=0.4466<br>P4=-0.4542<br>P5=-1.2533<br>P6=-1.3231<br>P7=1.4632			P3

	Dispersion model							
	**Estimate**	**SE**	**z-value**	**p-value**	**Estimate**	**SE**	**z-value**	**p-value**
Intercept	12.444	0.0539	23.064	$<2\times10^{-16}$	1.4359	0.0321	44.67	$<2\times10^{-16}$
C19	-0.411	0.1746	-2.355	0.0185	0.1439	0.4229	0.34	0.734
TD	-0.5138	0.2822	-1.821	0.0687	-0.3596	0.3514	-1.02	0.306

SE: Standard error; C19: COVID-19 infection; NHR: Nursing Home Residents; HCW: Health Care Workers; TD: third dose.

Table S4. Estimates of the means and probabilities of reaching the maximum antibody response in NHRs.

		$log\frac{\mu_{ij}}{1-\mu_{ij}} = 1.8449 + \beta_j - 0.7006 \times C19 - 1.4133 \times TD$				$\mu_{ij} = \frac{e^{1.8449+\beta_j-0.7006\times C19-1.4133\times TD}}{1+e^{1.8449+\beta_j-0.7006\times C19-1.4133\times TD}}$			
	b_j	*C19=1, TD=1*	*C19=1, TD=0*	*C19=0, TD=1*	*C19=0, TD=0*	*C19=1, TD=1*	*C19=1, TD=0*	*C19=0, TD=1*	*C19=0, TD=0*
P0	0.7245				2.5694				0.9289
P1	-0.0409		1.1034		1.8040		0.7509		0.8586
P2	-0.3635		0.7808		1.4814		0.6858		0.8148
P3	-0.2664		0.8779		1.5785		0.7064		0.8290
P4	-0.0855				1.7594				0.8531
P5	-0.0156		1.1287		1.8293		0.7556		0.8617
P6	0.1039	-0.1651	1.2482	0.5355	1.9488	0.4588	0.7770	0.6308	0.8753
P7	-0.0816	-0.3506	1.0627	0.3500	1.7633	0.4132	0.7432	0.5866	0.8536
	γ_j	$log\frac{P(Y_{ij}=0)}{1-P(Y_{ij}=0)} = 3.0564 + \gamma_j - 2.173 \times C19 + 5.152 \times TD$				$P(Y_{ij}=0) = \frac{e^{-3.0564+\gamma_j+2.173\times C.19+5.152\times TD}}{1+e^{-3.0564+\gamma_j+2.173\times C19+5.152\times TD}}$			
P0	0.7245	-0.2022							0.0370
P1	-0.0409	0.7492		-0.1342			0.4665		0.0905
P2	-0.3635	0.3735		-0.5099			0.3752		0.0640
P3	-0.2664	0.0673		-0.8161			0.3066		0.0479

P4	-0.0855	-0.2771							0.0344
P5	-0.0156	-0.2851		-1.1685			0.2371		0.0342
P6	0.1039	-0.2438	4.0248	-1.1272	1.8518	0.9824	0.2447	0.8643	0.0356
P7	-0.0816	-0.1077	4.1609	-0.9911	1.9879	0.9846	0.2707	0.8795	0.0405

C19: COVID-19; NHR; TD: third dose.

Table S5. Estimates of the means and probabilities of reaching the maximum antibody response in HCWs.

		$log\frac{\mu_{ij}}{1-\mu_{ij}} = 0.9321 + \beta_j - 1.8241 \times C19 - 0.5337 \times TD$				$\mu_{ij} = \frac{e^{0.9321+\beta_j-1.8241\times C19-0.5337\times TD}}{1+e^{0.9321+\beta_j-1.8241\times C19-0.5337\times TD}}$			
	b_j	*C19=1, TD=1*	*C19=1, TD=0*	*C19=0, TD=1*	*C19=0, TD=0*	*C19=1, TD=1*	*C19=1, TD=0*	*C19=0, TD=1*	*C19=0, TD=0*
P0	1.6582				2.5903				0.9302
P1	-0.927		-1.819		0.0051		0.1396		0.5013
P2	-0.4622				0.4699				0.6154
P3	-0.4957		-1.3877		0.4364		0.1998		0.6074
P4	-0.2591		-1.1511		0.673		0.2403		0.6622
P5	-0.0191		-0.9111		0.913		0.2868		0.7136
P6	0.352	-1.0737	-0.54	0.7504	1.2841	0.2547	0.3682	0.6793	0.7831
P7	0.1369	-1.2888	-0.7551	0.5353	1.069	0.2161	0.3197	0.6307	0.7444
	$log\frac{P(Y_{ij}=0)}{1-P(Y_{ij}=0)} = -2.766 + \gamma_j + 4.3071 \times C19 + 4.2206 \times TD$					$P(Y_{ij}=0) = \frac{e^{-2.766+\gamma_j+4.3071\times C19+4.2206\times TD}}{1+e^{-2.766+\gamma_j+4.3071\times C19+4.2206\times TD}}$			
	γ_j	*C19=1, TD=1*	*C19=1, TD=0*	*C19=0, TD=1*	*C19=0, TD=0*	*C19=1, TD=1*	*C19=1, TD=0*	*C19=0, TD=1*	*C19=0, TD=0*

P0	-1.812				-4.578				0.0102
P1	2.4209		3.962		-0.3451		0.9813		0.4146
P2	0.8448				-1.9212				0.1277
P3	0.4466		1.9877		-2.3194		0.8795		0.0895
P4	-0.4542		1.0869		-3.2202		0.7478		0.0384
P5	-0.1253		1.4158		-2.8913		0.8047		0.0526
P6	-1.3231	4.4386	0.218	0.1315	-4.0891	0.9883	0.5543	0.5328	0.0165
P7	1.4632	7.2249	3.0043	2.9178	-1.3028	0.9993	0.9528	0.9487	0.2137

Notes: C19: COVID19 infection; TD: Third dose

Table S6. Estimates of the "minimal" regression models for the mean with random intercepts, for the probability of reaching the maximum antibody response, and for the precision parameter for NHR group.

		**NHR**		**HCW**	
		**Conditional model**			
		**Estimate**	**p-value**	**Estimate**	**p-value**
	C19	-0.802	2.07×10^{-6}	-1.959	9.19×10^{-12}
	TD	-2.086	5.25×10^{-9}	-0.664	4.33×10^{-2}
	BMI<18.5	0.476	3.70×10^{-4}		
	BMI>25	0.231	0.044	-0.106	0.200
	Age	0.022	$<2\times10^{-16}$	0.021	$<2\times10^{-16}$
	Gender	0.082	0.474	0.039	0.437
	BMI>25*Gender	-0.097	0.600	0134	0.230
	Random intercepts	P0=0.4907	P1=-0.3408	P0=1.2999	P1=-0.9925
		P2=-0.6017	P3=-0.4459	P2=-0.5409	P3=-0.5281
		P4=-0.1832	P5=0.0562	P4=-0.286	P5=0.01156
		P6=0.3768	P7=0.1426	P6=0.5059	P7=-0.2920
		**Zero-Inflaction model**			
		**Estimate**	**p-value**	**Estimate**	**p-value**

	Intercept	-1.177	0.231	0.5047	0.1791
	C19	2.427	2.74×10^{-14}	4.163	$<2\times10^{-16}$
	TD	5.986	3.4×10^{-16}	5.859	$<2\times10^{-16}$
	BMI<18.5	-0.378	0.459		
	BMI>25	-0.279	0.385	-0.251	0.452
	Age	-0.015	0.194	-2.64×10^{-2}	0.001
	Gender	-49.1	0.067	-0.142	0.450
	Day	-0.012	0.005	-0.030	$<2\times10^{-16}$
	Day^2	2.21×10^{-5}	0.135	7.05×10^{-5}	6.77×10^{-9}
	BMI>25*Gender	-0.1699	0.739	-0.209	0.640
		**Dispersion model**			
		**Estimate**	**p-value**	**Estimate**	**p-value**
	C19	-0.678	2.78E-04	-0.477	0.264
	TD	-1.764	5.29E-08	-0.913	0.012
	BMI<18.5	1.139	5.74×10^{-9}		
	BMI>25	0.107	0.398	-0.106	0.357
	Age	0.010	$<2\times10^{-16}$	0.023	$<2\times10^{-16}$
	Gender	0.181	0.168	0.103	0.155
	Day	0.005	$3.8\times10^{-}11$	0.004	$<2\times10^{-16}$
	BMI>25*Gender	-0.024	0.8921	-0.207	0.178
AIC		-1387.4		-380.6	

Notes: C19: COVID-19 infection; HCWs: Health Care Workers; NHRs: Nursing Home Residents; TD: third dose.
